# Comparative efficacy of femoral neck system vs. cannulated compression screws in young patients with femoral neck fractures: a systematic review and meta-analysis

**DOI:** 10.3389/fsurg.2025.1626320

**Published:** 2025-10-09

**Authors:** Weishuai Zhang, Nannan Yang, Xianyou Zhu, Xuchao Lu, Jian Cheng

**Affiliations:** 1 The Fifth Ward of Orthopedics Department, Kaifeng People’s Hospital, Kaifeng, Henan Province, China; 2 Department of Pain, Kaifeng People’s Hospital, Kaifeng, Henan Province, China; 3 Department of Orthopedics, Xuzhou Central Hospital, Xuzhou, Jiangsu Province, China

**Keywords:** femoral neck fracture, femoral neck system, cannulated compression screws, internal fixation, meta-analysis, young adults

## Abstract

**Background:**

The optimal method for internal fixation of femoral neck fractures in younger individuals remains a subject of debate. This meta-analysis systematically evaluates and compares the clinical outcomes of the femoral neck system (FNS) and cannulated compression screws (CCSs) within this demographic.

**Methods:**

A comprehensive literature search was conducted across the Cochrane Library, PubMed, Web of Science, and Embase databases, covering studies from their inception through March 2024. The search targeted cohort studies that compared FNS (*n* = 265) and CCSs (*n* = 326) in patients aged 14–65 years with femoral neck fractures. The methodological quality of the studies was appraised using the Newcastle–Ottawa Scale. Statistical analyses were executed using RevMan 5.4, with results presented as standardized mean differences (SMDs) or weighted mean differences (WMDs), accompanied by 95% confidence intervals (CIs).

**Results:**

The analysis incorporated nine high-quality cohort studies involving 591 patients who underwent surgical procedures for femoral neck fractures. Of these patients, 265 were treated with the femoral neck system (FNS), while 326 were treated with CCSs. Meta-analysis revealed that, compared to CCS, FNS was associated with a significantly shorted fracture healing time (SMD = 16.30, 95% CI: 3.79–28.82, *P* < 0.001), decreased intraoperative fluoroscopy usage (WMD) = −8.14, 95% CI: −9.82 to −6.46, *P* < 0.001), and higher Harris hip scores at the final follow-up (WMD = −3.43, 95% CI: −4.08 to −2.77, *P* < 0.001). In addition, the FNS group exhibited a lower incidence of postoperative complications, including urinary tract infections, venous thromboembolism, non-union, screw loosening, and femoral head necrosis [risk ratio (RR) = 1.05, 95% CI: 0.92–1.19, *P* = 0.50]. However, the FNS was associated with a longer surgical incision (WMD = 0.84, 95% CI: 0.55–1.13, *P* < 0.001) and increased intraoperative blood loss (WMD = 16.30, 95% CI: 3.79–28.82, *P* = 0.01). The analysis revealed no statistically significant differences between the two techniques in terms of operation duration (WMD = −4.88, 95% CI: −12.25 to 2.48, *P* = 0.19), length of hospital stay (WMD = 0.10, 95% CI: −0.20 to 0.40, *P* = 0.52), or the excellent-to-good rate at the final follow-up (RR = 1.05, 95% CI: 0.92–1.19, *P* = 0.50).

**Conclusions:**

The femoral neck system (FNS) may present potential benefits in specific outcomes, notably expedited healing and enhanced functional rehabilitation. The results of this study advocate for the consideration of the FNS as a preferred treatment option for active patients, where minimizing radiation exposure and optimizing long-term outcomes are prioritized, despite its slightly greater invasiveness.

## 1 Introduction

Femoral neck fractures represent a significant orthopedic issue, accounting for approximately 53% of all hip fractures (1). Due to the distinct anatomical and biomechanical properties of the femoral neck, conservative treatment poses considerable risks of femoral head necrosis, establishing surgical intervention as the standard approach (2). While hip arthroplasty is typically favored in patients aged 65 years and older due to its potential for facilitating rapid functional recovery, internal fixation remains the preferred approach in younger patients, given the limited lifespan of prosthetic implants. The choice of the most appropriate internal fixation method remains a subject of ongoing debate. Current clinical practice employs a variety of techniques, including cannulated compression screws (CCSs), femoral neck systems (FNSs), dynamic hip screws, locking plates, and intramedullary nails. Among these, the CCS approach has been the predominant choice, while the FNS was initially introduced in China in 2019 (3). Although both the FNS and CCSs have demonstrated clinical efficacy in treating femoral neck fractures, existing studies present conflicting results concerning operative duration, fracture healing time, final Harris hip scores, and postoperative complications, such as femoral head necrosis, wound infection, delayed union, non-union, and femoral neck shortening (4–8). This systematic evidence synthesis seeks to compare CCS and FNS through a meta-analysis of contemporary clinical studies, thereby providing clinicians with updated guidance for surgical decision-making.

## 2 Literature search strategy

**Figure 1:**
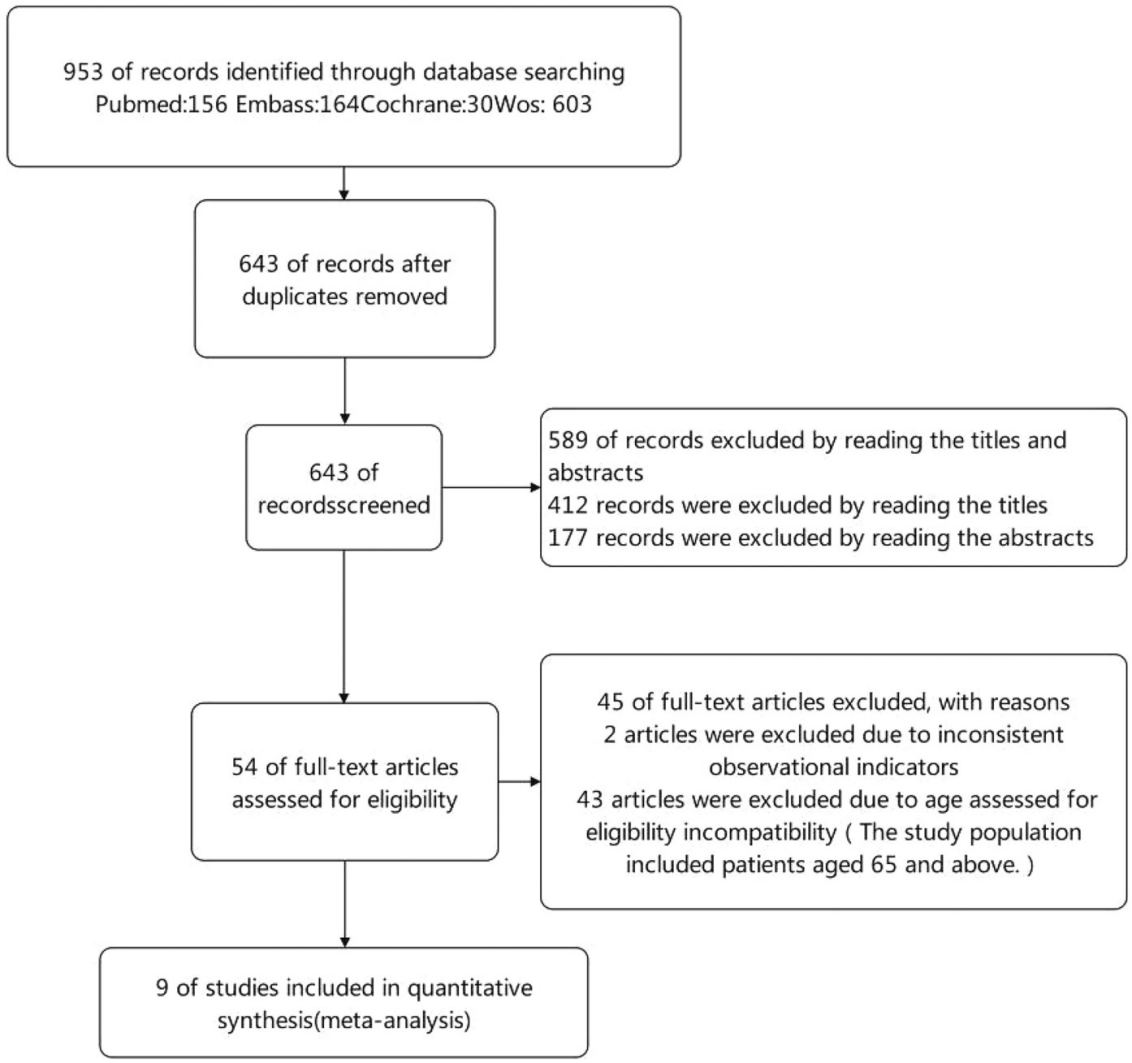
Study flow diagram.

A comprehensive literature search was conducted utilizing the following search terms: “femoral neck fractures,” “Femoral Neck Fractures,” “Femoral Neck Fracture,” “Femur Neck Fractures,” and “Femur Neck Fracture,” in conjunction with “hollow screw,” “cannulated screws,” “hollow bolt,” and “cannulated screw,” as well as “Femoral Neck System” or “femoral neck screw system.” This search was performed across several databases, including the Cochrane Library, PubMed, Web of Science, and Embase, covering the period from the inception of each database to 6 August 2024. The search strategy did not intentionally exclude studies published in languages other than English. The detailed search methodology is depicted in Figure 1.

The precise search strategy employed was: (Femoral Neck System) OR (Femoral Neck System) OR (FNS) AND (cannulated screw) OR (cannulated screws) OR (hollow screw) OR (cannulated compression screw) AND (Femoral Neck System) OR (Femoral Neck System) OR (FNS).

In accordance with PRISMA (Preferred Reporting Items for Systematic Reviews and Meta-Analyses) guidelines, the literature screening process was conducted in four distinct stages. Initially, 953 studies were identified through database searches. After removing duplicates, 643 progressed to the screening stage. Of these, 589 were excluded after reviewing titles and abstracts that did not meet the inclusion criteria. The remaining 54 studies were subjected to an eligibility assessment. Following a comprehensive full-text evaluation, 45 studies were excluded—2 due to inconsistent observational metrics and 43 because they included patients older than 65 years. Consequently, nine high-quality cohort studies were selected for inclusion in the meta-analysis.

### 2.1 Inclusion and exclusion criteria

This systematic review adhered rigorously to the PRISMA guidelines (9). The inclusion criteria were (1) young and middle-aged patients aged 18–65 years with surgically treated femoral neck fractures, (2) comparative studies assessing the fixation techniques of FNS vs. CCS, and (3) original research presenting quantifiable outcomes, including randomized controlled trials, cohort studies, or case–control studies. The exclusion criteria were (1) studies consisting of patients older than 65 years, (2) studies involving hybrid fixation methods that combined the FNS/CCSs with other devices, and (3) non-comparative study designs, such as case reports, reviews, conference abstracts, or studies with incomplete outcome data.

### 2.2 Data extraction and processing

The primary outcome measures included surgical duration, fracture healing duration, and the incidence of complications. The secondary outcome measures encompassed incision length, intraoperative blood loss, length of hospital stay, Harris hip score at the final follow-up, the number of intraoperative fluoroscopy sessions, and the rates of excellent and good outcomes at the final follow-up. Two independent researchers conducted literature screening and data extraction. In instances where discrepancies arose between the two researchers regarding the extracted data, a third researcher was consulted to perform comprehensive cross-checks until consensus was achieved. Literature screening was executed using EndNote X9 (Clarivate Analytics, UK) software, which facilitated the removal of duplicates. Studies not meeting the inclusion criteria were excluded after a review of titles and abstracts. The remaining articles underwent a full-text review, leading to the selection of studies appropriate for a qualitative systematic review and subsequent meta-analysis.

### 2.3 Literature quality assessment

The quality of cohort studies was assessed utilizing the Newcastle–Ottawa Scale (NOS) (10). The evaluation criteria encompassed three domains: selection of the study population, comparability between groups, and assessment of exposure factors. The maximum score of the scale is 9 points. Studies scoring ≥7 were considered high-quality, those scoring between 5 and 6 were classified as medium-quality, and those scoring <5 were deemed low-quality. Two researchers independently conducted the evaluations. In instances of disagreement, a third researcher was consulted to perform an additional evaluation until consensus was attained.

### 2.4 Outcome measures: baseline indicators

The following baseline indicators were evaluated: first author, year of publication, number of cases, mean age, surgical technique, duration of follow-up, Garden classification of femoral neck fractures, and the number of outcome measures (refer to Table 1). The primary outcome measures identified were surgical duration, time to fracture healing, and the incidence of complications. The secondary outcome measures encompassed incision length, intraoperative blood loss, duration of hospital stay, Harris hip score at the final follow-up, number of intraoperative fluoroscopy sessions, and rates of excellent and good outcomes at the final follow-up.

**Table 1:** Characteristics of included articles.

Included study	Number of cases	Average age	FNS	CCS	Garden classification (Ⅰ/Ⅱ/Ⅲ/Ⅳ)		Follow-up time (months)	Outcome indicator
	FNS/CCS	FNS/CCS	Male/Female	Male/Female	FNS	CCS	FNS/CCS	
He et al. (11)	33/36	50.61/47.58	18/15	22/14	1/8/19/5	2/9/20/5	16.91 ± 3.01	①③④⑤⑥⑦⑧⑨
Hu et al. (12)	20/24	50.45/50.46 ± 9.26	8/12	14/12	0/6/8/6	4/6/7/7	>12	① ⑦⑧
Kenmegne et al. (13)	56/58	58.20/40.45	29/27	34/24	3/13/37/0	0/12/41/5	27 ± 2.07	①②③④⑤⑥⑦⑧⑨
Lu et al. (14)	28/30	14.5/14.3	19/9	22/8	—	—	16.3/17.0	①③④⑥⑧⑨
Wang et al. (15)	30/30	41.83/41.29 ± 13.48	18/12	17/13	—	—	6.0	①③④⑤⑥⑦⑧⑨
Yan et al. (16)	24/58	52/49	14/10	38/20	0/4/12/8	2/10/32/14	7.3/13.6	①④⑥⑦
Yang et al. (17)	28/31	51/49	17/11	17/14	0/5/12/11	0/4/16/11	8.7/9.1	①②④⑤⑥⑦
Zhang et al. (18)	25/27	45.32/49.3	14/11	16/11	0/2/15/8	0/2/16/9	15.04/16.19	①②④⑥⑦
Zhao et al. (19)	21/32	46.4/45.1	12/9	15/17	1/0/3/17	2/0/5/25	19/21	① ④⑥⑧⑨

## 3 Statistical analysis

The data were analyzed utilizing RevMan 5.4 software. Heterogeneity among the included studies was evaluated using the *Q*-test with a significance level of *α* = 0.1. In addition, the *I*^2^ statistic was used to quantitatively assess the degree of heterogeneity. A fixed-effects model was employed for the meta-analysis when *P* > 0.1 or *I*^2^ < 50%, indicating low heterogeneity. Conversely, when *P* ≤ 0.1 or *I*^2^ ≥ 50%, high heterogeneity was inferred, prompting further analysis to identify its sources. Following the exclusion of evident clinical heterogeneity, a random-effects model was applied for the meta-analysis. The significance level (*α*) was set at 0.05. In cases of significant clinical heterogeneity, subgroup or sensitivity analyses were conducted. If these analyses were not feasible, a descriptive analysis was performed. For continuous variables, the weighted mean difference (WMD) or standardized mean difference (SMD) was used as the pooled statistic, while for dichotomous variables, the risk ratio (RR) was employed.

## 4 Results

### 4.1 Literature search results

A total of 953 articles were identified through the literature search. After applying the specified inclusion and exclusion criteria, a thorough screening resulted in nine articles being selected for final analysis. These included nine high-quality cohort studies (11–19) involving 591 patients who underwent surgical treatment for femoral neck fractures. Of these patients, 265 were treated with the femoral neck system (FNS), while 326 received cannulated compression screw (CCS treatment. Patient ages ranged from 41.2 to 57.6 years, and fractures were classified as Garden types I–IV. The follow-up period lasted between 6 and 24 months.

### 4.2 Quality assessment of the literature

All nine included studies were cohort studies, encompassing 591 young and middle-aged patients who underwent internal fixation for femoral neck fractures. Study quality was assessed using the NOS. Of these, eight studies were rated as high-quality, while one was classified as medium-quality (see Table 2).

**Table 2:** NOS evaluation of literature quality in cohort studies.

Included studies	Selection of the study population	Comparability between groups	Outcome measurement	Total score
He et al. (11)	3	1	3	7
Hu et al. (12)	3	1	2	6
Kenmegne et al. (13)	3	2	2	7
Lu et al. (14)	3	2	2	7
Wang et al. (15)	3	2	3	8
Yan et al. (16)	3	2	2	7
Yang et al. (17)	3	2	2	7
Zhang et al. (18)	3	2	2	7
Zhao et al. (19)	3	2	2	7

### 4.3 Meta-analysis results

#### 4.3.1 Operation time

**Figure 2:**
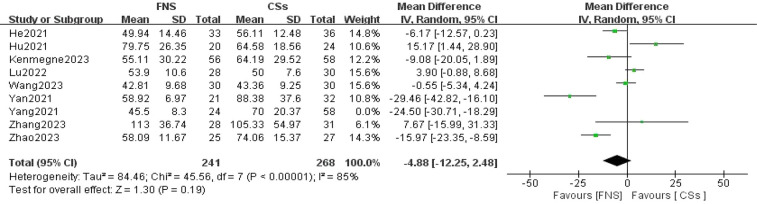
Forest plot comparing the difference in operation times between the FNS and CCS groups indicates that the operation time for the FNS group was shorter than that for the CCS group.

Nine studies (11–19) were included in the analysis comparing operation time. The forest plot indicated a statistically significant difference between the two groups. However, substantial heterogeneity was observed (*I*^2^ = 92%, *P* = 0.009). To identify the source of this heterogeneity, a sensitivity analysis was performed. Excluding one study (17) resulted in the greatest reduction in heterogeneity, decreasing *I*^2^ to 85%. Despite this adjustment, the difference between the two groups remained statistically non-significant, and substantial heterogeneity persisted. A thorough examination of the full texts of the included studies suggested that the heterogeneity could be attributed to variations in the technical proficiency levels of surgeons across different regions and differences in the sample sizes of the studies. A meta-analysis using a random-effects model indicated that there was no statistically significant difference in operation time between the FNS and CCS groups [WMD = −4.88, 95% confidence interval (CI): −12.25 to 2.48, *P* = 0.19; Figure 2].

#### 4.3.2 Length of surgical incisions

**Figure 3:**
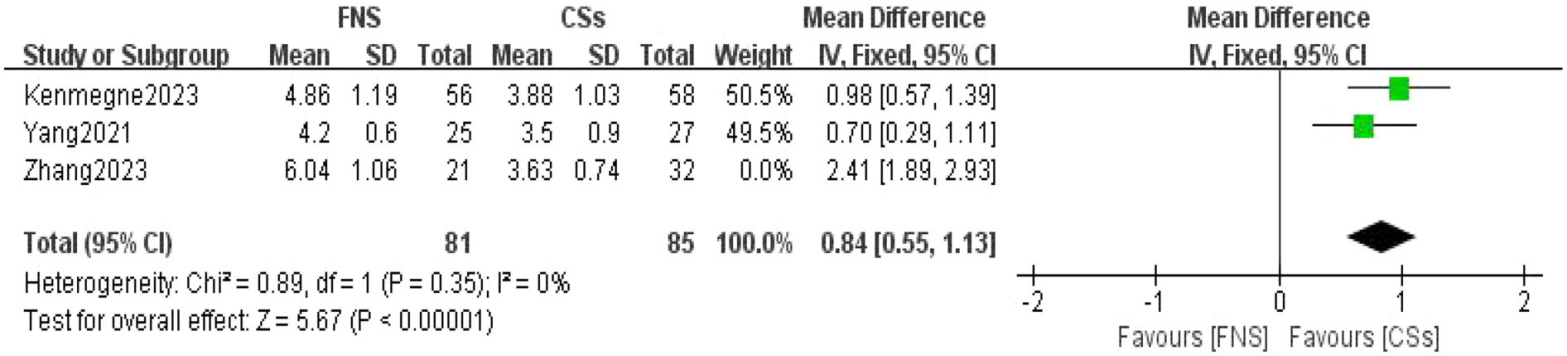
Forest plot comparing the lengths of surgical incision between the FNS and CCS groups indicates that the surgical incision length was longer in the FNS group than in the CCS group.

Three studies (13, 17, 18) were included in the analysis comparing the length of surgical incisions. The forest plot revealed significant statistical heterogeneity (*I*^2^ = 93%, *P* < 0.001). To identify the source of this heterogeneity, a sensitivity analysis was performed. The exclusion of one study (18) resulted in a substantial reduction in heterogeneity (*I*^2^ = 0%), suggesting that this particular study was primarily responsible for the observed heterogeneity. A meta-analysis using a fixed-effects model demonstrated a statistically significant difference in the length of surgical incisions between the FNS and CCS groups [mean difference (MD) = 0.84, 95% CI: 0.55–1.13, *P* < 0.001; Figure 3].

#### 4.3.3 Intraoperative fluoroscopy usage

**Figure 4:**
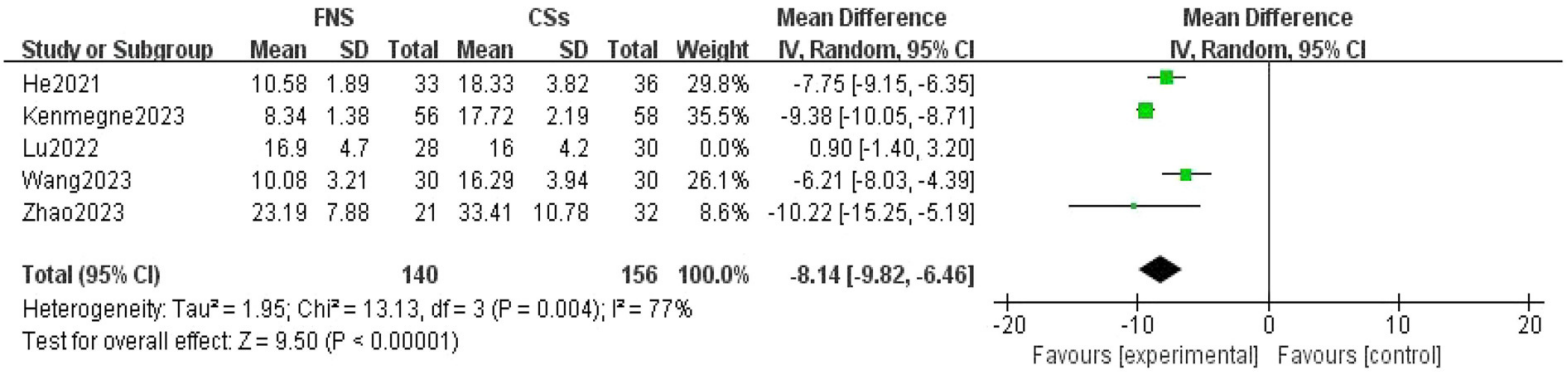
Forest plot comparing the intraoperative fluoroscopy times between the FNS group and the CCS group indicates that the FNS group had fewer intraoperative fluoroscopy times than the CCS group.

Five studies (11, 13–15, 19) were included in the analysis comparing the frequency of intraoperative fluoroscopy usage. The forest plot revealed significant statistical heterogeneity (*I*^2^ = 95%, *P* < 0.001). To identify the source of this heterogeneity, a sensitivity analysis was conducted. Exclusion of one study (14) resulted in the most pronounced reduction in heterogeneity, decreasing the *I*^2^ value to 77%. Despite the persistence of substantial heterogeneity, the consistency of the results suggested the robustness of the findings. A thorough review of the full-text articles indicated that this heterogeneity was primarily attributable to variations in operator proficiency levels. A meta-analysis utilizing a random-effects model demonstrated a statistically significant difference in the number of intraoperative fluoroscopy sessions between the femoral neck system (FNS) and cannulated compression screw (CCS) groups (MD = −8.14, 95% CI: −9.82 to −6.46, *P* < 0.001; Figure 4).

#### 4.3.4 Intraoperative blood loss

**Figure 5:**
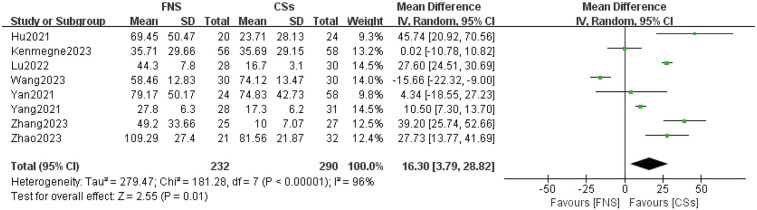
Forest plot comparing the amounts of intraoperative blood loss between the FNS and CCS groups indicates that there was more intraoperative blood loss in the FNS group than in the CCS group.

Eight studies were analyzed to compare intraoperative blood loss (12–19). The forest plot revealed significant statistical heterogeneity (*I*^2^ = 96%, *P* < 0.001). To identify the source of this heterogeneity, a sensitivity analysis was conducted. Exclusion of any single study did not significantly impact the heterogeneity or alter the overall results. A comprehensive review of the full texts of the included studies suggested that the heterogeneity was due to variations in surgical techniques and outcome measures. A meta-analysis utilizing a random-effects model indicated that the difference in intraoperative blood loss between the FNS and CCS groups was statistically significant (MD = 16.30, 95% CI: 3.79–28.82, *P* = 0.01; Figure 5).

#### 4.3.5 Length of hospital stay

**Figure 6:**
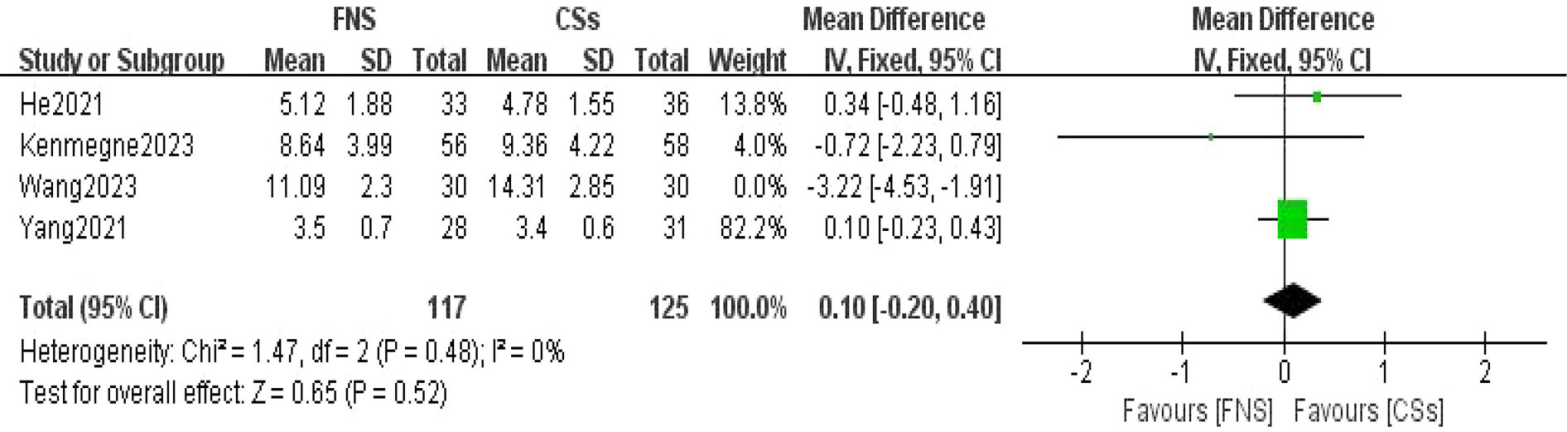
Forest plot comparing the lengths of hospital stay between the FNS and CCS groups indicates that there was no significant difference in the length of hospital stay between the two groups.

Four studies (11, 13, 15, 17) were utilized to assess the length of hospital stay. The forest plot revealed significant statistical heterogeneity (*I*^2^ = 88%, *P* < 0.001). To identify the source of this heterogeneity, a sensitivity analysis was conducted. Exclusion of one particular study (15) resulted in the most notable reduction in heterogeneity, decreasing *I*^2^ to 0%. The stability of the results suggests that this specific study was the primary contributor to the observed heterogeneity. A meta-analysis employing a random-effects model indicated that the difference in length of hospital stay between the FNS and CCS groups was not statistically significant (MD = 0.10, 95% CI: −0.20 to 0.40, *P* = 0.52; Figure 6).

#### 4.3.6 Fracture healing time

**Figure 7:**
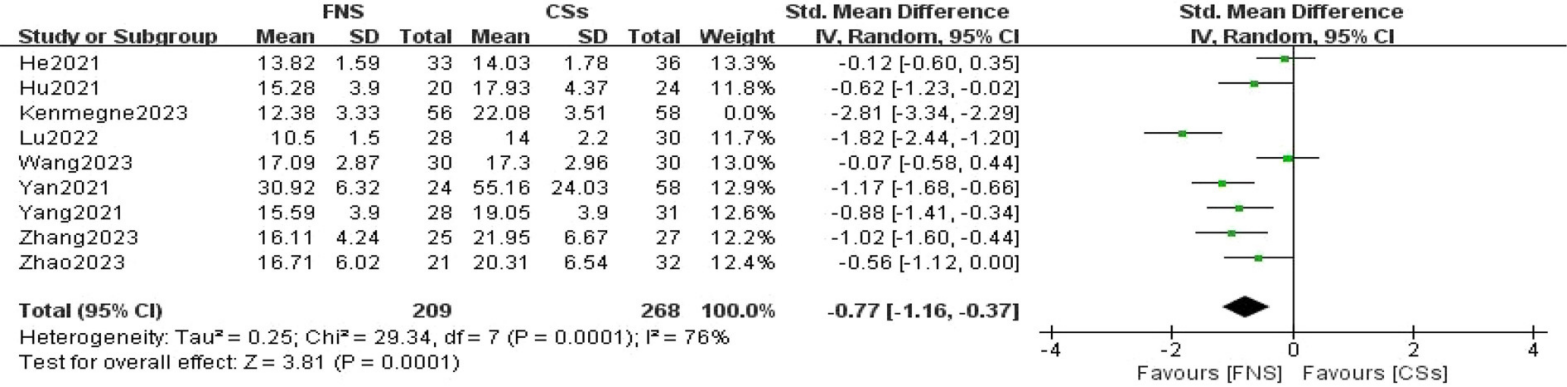
Forest plot comparing the fracture healing times of patients in the FNS and CCS groups indicates that the fracture healing time in the FNS group was shorter than in the CCS group.

Nine studies (11–19) were analyzed to compare fracture healing times. The forest plot revealed significant statistical heterogeneity (*I*^2^ = 90%, *P* < 0.001). To identify the source of this heterogeneity, a sensitivity analysis was conducted with precision. Notably, excluding one particular study resulted in the most significant reduction in heterogeneity, decreasing *I*^2^ to 76% (13). The robustness of the results strongly indicates that this study was the primary contributor to the observed heterogeneity, although the level of heterogeneity remained relatively high. A thorough review of the full texts revealed that substantial regional disparities among the studies, as well as variations in personnel and standards used to assess fracture healing time, were the primary sources of this heterogeneity. A meta-analysis conducted using a random-effects model demonstrated that the difference in postoperative fracture healing time between the FNS and CCS groups was statistically significant (SMD = 16.30, 95% CI: 3.79–28.82, *P* < 0.001; Figure 7).

#### 4.3.7 Harris hip score at the final follow-up

**Figure 8:**
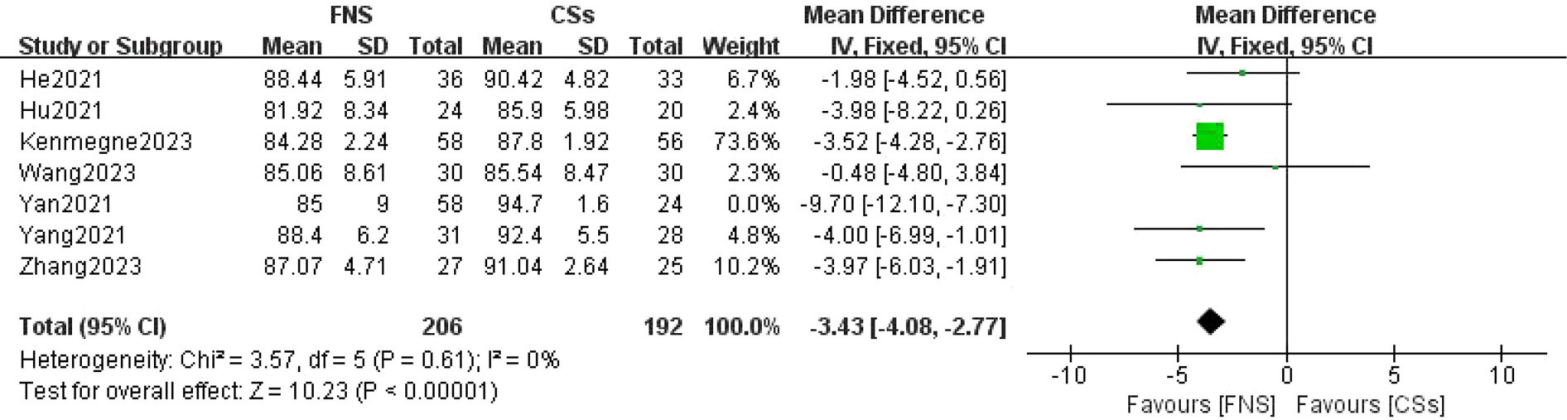
Forest plot comparing the Harris hip scores of patients in the FNS and CCS groups at the last follow-up indicates that the Harris hip score in the FNS group was higher than in the CCS group.

Seven studies (11–13, 15–18) were included in the analysis comparing Harris hip scores at the final follow-up. The forest plot revealed significant statistical heterogeneity (*I*^2^ = 79%, *P* < 0.001). To identify the source of this heterogeneity, a sensitivity analysis was conducted. The exclusion of one specific study resulted in the most pronounced reduction in heterogeneity, decreasing *I*^2^ to 0% (16). The stability of the results indicates that this particular study was the primary contributor to the observed heterogeneity. A meta-analysis utilizing a fixed-effects model demonstrated that the difference in Harris hip scores at the final follow-up between the FNS and CCS groups was statistically significant (WMD = −3.43, 95% CI: −4.08 to −2.77, *P* < 0.001; Figure 8).

#### 4.3.8 Complication rate

**Figure 9:**
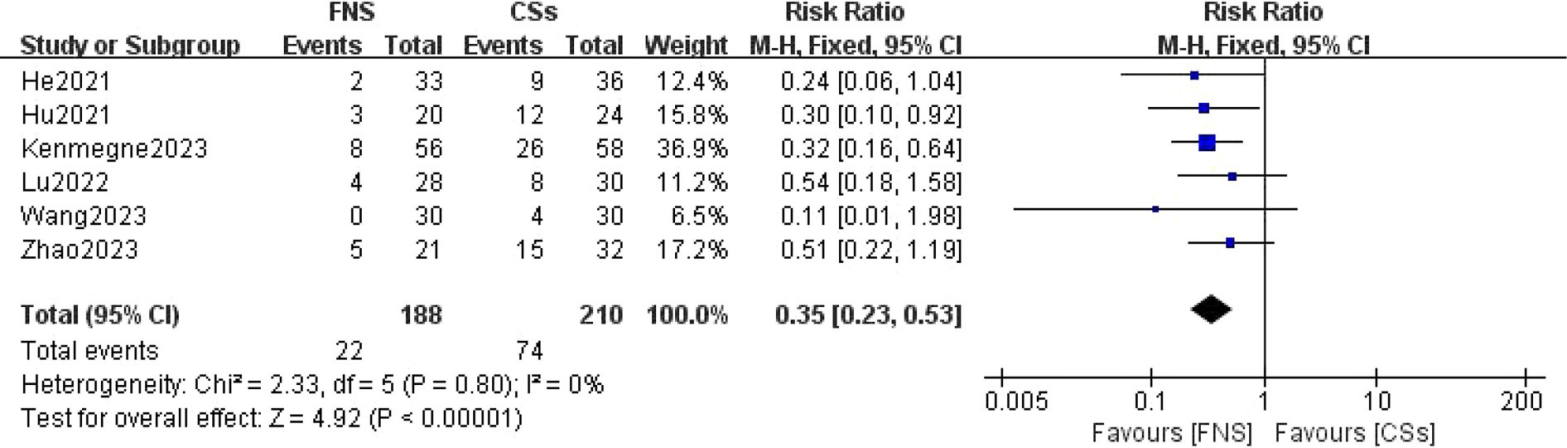
Forest plot comparing the postoperative complication rates between the FNS and CCS groups indicates that the postoperative complication rate in the FNS group was lower than in the CCS group.

A comprehensive analysis of six studies (11–15, 19) was conducted to compare complication rates. The forest plot indicated an absence of statistical heterogeneity (*I*^2^ = 0%, *P* = 0.80). Utilizing a fixed-effects model for the meta-analysis, a statistically significant difference in complication rates between the FNS and CCS groups was identified (RR = 0.35, 95% CI: 0.23–0.53, *P* < 0.001; Figure 9).

#### 4.3.9 Excellent-to-good rate at the final follow-up

**Figure 10:**
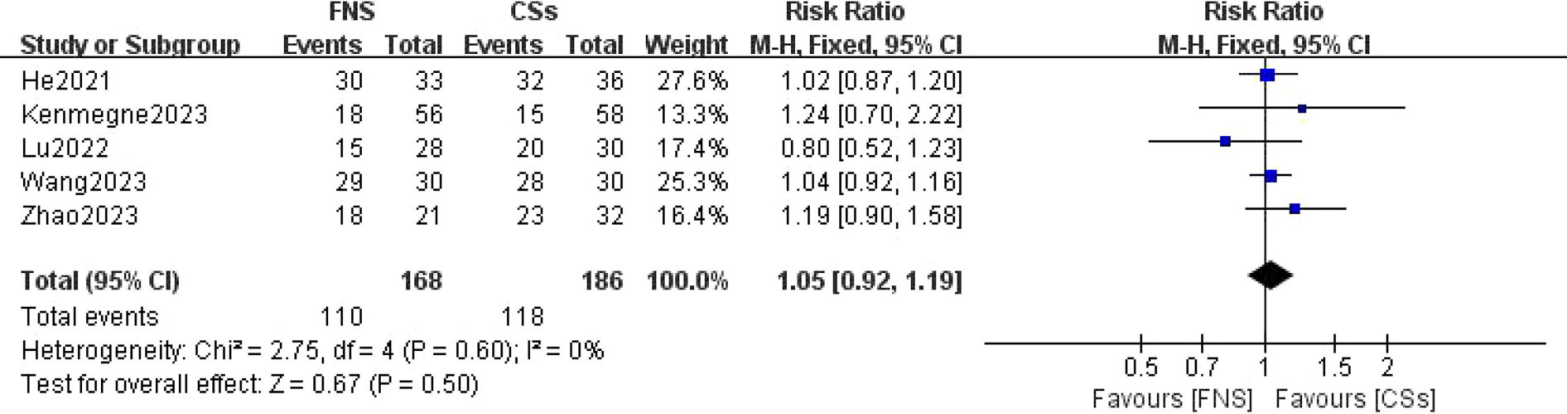
Forest plot comparing the excellent and good rates at the last follow-up between the FNS and CCS groups indicates that there was no significant difference in the excellent and good rates between the two groups.

Five studies (11, 13–15, 19) focused on comparing the excellent-to-good rates at the final follow-up. The forest plot clearly illustrated the lack of statistical heterogeneity, as indicated by an *I*^2^ value of 0% and a *P*-value of 0.60. A meta-analysis employing a fixed-effects model revealed that the difference in excellent-to-good rates between the FNS and CCS groups was not statistically significant (RR = 1.05, 95% CI: 0.92–1.19, *P* = 0.50; Figure 10).

### 4.4 Evaluation of publication bias

Funnel plots were constructed for operative time and fracture healing time as primary outcome measures. The asymmetrical distribution observed in these plots suggests the presence of potential publication bias. Notably, a significant proportion of the studies included in this analysis were conducted by researchers from China, which may contribute to and potentially amplify the observed bias. All funnel plots are provided as Supplementary Material.

## 5 Discussion

This meta-analysis provides substantial clinical insights from a comparative evaluation of the femoral neck system (FNS) and cannulated compression screw (CCS) techniques in the management of femoral neck fractures among young adults. The results indicate that the FNS outperforms CCS across various intraoperative and postoperative parameters, offering valuable guidance for clinical practice. Notably, the FNS group exhibited a significantly reduced frequency of intraoperative fluoroscopy sessions compared to the CCS group (MD = −8.14, 95% CI: −9.82 to −6.46, *P* < 0.001). This decrease in fluoroscopy usage can be attributed to the streamlined design of the FNS, which incorporates a single sliding screw and an anti-rotation side plate. Conversely, the CCS approach necessitates the use of multiple screws, complicating the positioning and fixation process. Consequently, surgeons can achieve precise screw placement more efficiently with the FNS, thereby diminishing reliance on fluoroscopic guidance (21). Furthermore, the design of the FNS is more compatible with the anatomical configuration of the proximal femur and femoral neck, requiring fewer adjustments in fluoroscopy angles and positioning compared to the CCS (22). In the context of fracture healing, the FNS group demonstrated a significantly shorter healing duration than the CCS group (SMD = 16.30, 95% CI: 3.79–28.82, *P* < 0.001). This advantage is primarily attributed to the biomechanical design of the FNS system, which integrates a single slip screw with an anti-rotation side plate. This configuration effectively minimizes rotational stress and shear forces, promoting more stable fixation, reducing fretting at the fracture site, mitigating inflammatory responses, and enhancing the overall healing process (23). At the final follow-up, the Harris score for hip function was significantly higher in the FNS group than in the CCS group (WMD = −3.43, 95% CI: −4.08 to −2.77, *P* < 0.001). This finding underscores the superior recovery of hip function associated with the FNS, which effectively maintains fracture alignment and provides stronger anchorage than hollow screws. Patients treated with the femoral neck system (FNS) are able to bear weight and mobilize earlier, which contributes to a reduced incidence of postoperative complications such as screw loosening and bone non-union. These factors are essential for achieving stable functional recovery of the hip joint. In addition, the streamlined pain management and rehabilitation processes associated with the FNS lead to improved functional outcomes during follow-up evaluations. The incidence of postoperative complications, including femoral head necrosis, incision infections, delayed union, fracture non-union, femoral neck shortening, and thigh pain, was significantly lower in the FNS group than in the cannulated compression screw (CCS) group [RR = 0.35, 95% CI: 0.23–0.53, *P* < 0.001]. The distinctive fixation method employed by the FNS facilitates uniform stress distribution, thereby reducing the risks of screw loosening and fracture displacement (24). This mechanical stability is vital for decreasing the incidence of bone non-union and femoral head necrosis. Furthermore, enhanced biomechanical stability diminishes the likelihood of postoperative local inflammation and osteonecrosis, resulting in a lower overall complication rate among patients (20). However, despite its advantages, the FNS is associated with a significantly longer incision (MD = 0.84, 95% CI: 0.55–1.13, *P* < 0.001) and increased intraoperative blood loss compared to CCSs (MD = 16.30, 95% CI: 3.79–28.82, *P* = 0.01). The necessity for a longer incision for the FNS arises from the need to adequately expose the surgical site to fix the femoral lateral plate, which involves removal of proximal femoral soft tissue and consequently results in increased intraoperative bleeding. Although these factors could potentially contribute to delayed healing and an elevated risk of infection, our study did not reveal a significant increase in these complications. Furthermore, no substantial differences were observed between FNS and CCS groups with respect to operation time (WMD = −4.88, 95% CI: −12.25 to 2.48, *P* = 0.19), length of hospital stay (MD = 0.10, 95% CI: −0.20 to 0.40, *P* = 0.52), or the excellent-to-good rate at the final follow-up [risk ratio (RR) = 1.05, 95% CI: 0.92–1.19, *P* = 0.50]. This observation may indicate minimal differences in surgical techniques and patient recovery trajectories. Factors including surgeon experience, intraoperative management skills, and postoperative care are likely to exert a similar influence on both procedures, potentially diminishing the inherent advantages of fixation systems. Furthermore, the comparable rates of excellent and good outcomes at the final follow-up imply that, although the femoral neck system (FNS) demonstrates certain short-term benefits, patients treated with the cannulated compression screw (CCS) approach can attain favorable functional recovery over time through effective rehabilitation.

### 5.1 Limitations of the study

This research has several limitations that warrant acknowledgment. First, a significant shortcoming of this paper is the absence of preregistration, as we were unaware of the registration requirements during the design phase of this systematic review. Second, the literature search was confined to four widely utilized English-language databases, potentially leading to the exclusion of studies published in other languages. It is also important to note that substantial research on the femoral neck system (FNS) has been conducted by Chinese researchers in recent years, which may introduce reporting bias.

To enhance the generalizability of findings related to the FNS, future multicenter international studies, particularly those incorporating data from European and American populations, are essential. Notably, some of the studies included exhibited significant heterogeneity in baseline characteristics such as patient age, which may have contributed to the observed variability. Moreover, certain secondary outcomes were not included in the meta-analysis, limiting the comprehensiveness of our conclusions. All studies reviewed were cohort studies with relatively small sample sizes, potentially affecting the generalizability of the results and contributing to the high heterogeneity observed in the outcomes. In addition, the follow-up durations in some studies were short and varied considerably, limiting the ability to fully assess long-term outcomes. These limitations should be considered when interpreting the findings and their implications for clinical practice. Future validation through randomized controlled trials, which provide higher levels of evidence, will be necessary.

## 6 Conclusion

In conclusion, compared to cannulated compression screws (CCS), the femoral neck system (FNS) presents distinct advantages in managing femoral neck fractures among young and middle-aged adults. These advantages include reduced operation time, decreased intraoperative fluoroscopy exposure, accelerated fracture healing, and a lower incidence of postoperative complications, such as femoral head necrosis, incision infection, delayed union, non-union, femoral neck shortening, and thigh pain. These findings indicate that the FNS holds promise for broader clinical adoption and application. Nonetheless, in clinical practice, the benefits of the FNS should be thoroughly assessed in conjunction with patient-specific variables, such as patient age, to ensure optimal outcomes.

## Data Availability

The original contributions presented in the study are included in the article/Supplementary Material, further inquiries can be directed to the corresponding author.
